# Outcomes linked to eligibility for stem cell transplantation trials in diffuse cutaneous systemic sclerosis

**DOI:** 10.1093/rheumatology/keab604

**Published:** 2021-07-26

**Authors:** Julia Spierings, Svetlana I Nihtyanova, Emma Derrett-Smith, Kristina E N Clark, Jacob M van Laar, Voon Ong, Christopher P Denton

**Affiliations:** 1 Division of Medicine, Department of Inflammation, Centre for Rheumatology and Connective Tissue Diseases, Royal Free and University College Medical School, University College London, London, UK; 2 Department of Rheumatology and Clinical Immunology, University Medical Centre Utrecht, Utrecht, The Netherlands

**Keywords:** SSc, diffuse scleroderma, autologous hematopoietic stem cell transplantation, clinical trials, immunosuppressive agents, survival analysis, event-free survival

## Abstract

**Objectives:**

The aim of this study was to explore outcomes in a cohort of dcSSc patients fulfilling eligibility criteria for stem cell transplantation (SCT) studies but receiving standard immunosuppression.

**Methods:**

From a large single-centre dcSSc cohort (*n* = 636), patients were identified using the published SCT trials’ inclusion criteria. Patients meeting the trials’ exclusion criteria were excluded.

**Results:**

Of the 227 eligible patients, 214 met the inclusion criteria for ASTIS (Autologous Stem Cell Transplantation International Scleroderma), 82 for SCOT (Scleroderma: Cyclophosphamide Or Transplantation) and 185 for the UPSIDE (UPfront autologous haematopoietic Stem cell transplantation vs Immunosuppressive medication in early DiffusE cutaneous systemic sclerosis) trial, and 66 were excluded based on age >65 years, low diffusing capacity of the lungs for carbon monoxide (DLco), pulmonary hypertension or creatinine clearance <40 ml/min. The mean follow-up time was 12 years (s.d. 7). Among the eligible patients, 103 (45.4%) died. Survival was 96% at 2 years, 88% at 5 years, 73% at 10 years and 43% at 20 years. Compared with this ‘SCT-eligible’ cohort, those patients who would have been excluded from SCT trials had a worse long-term survival (97% at 2 years, 77% at 5 years, 52% at 10 years and 15% at 20 years, log rank *P* < 0.001). Excluded patients also had a significantly worse long-term event-free survival. Hazard of death was higher in patients with higher age at onset [hazard ratio (HR) 1.05, *P* < 0.001], higher ESR at baseline (HR 1.01, *P* = 0.025) and males (HR 2.12, *P* = 0.008).

**Conclusion:**

SCT inclusion criteria identify patients with poor outcome despite current best practice treatment. Patients meeting the inclusion criteria for SCT but who would have been excluded from the trials because of age, pulmonary hypertension, poor kidney function or DLco <40% had worse outcomes.

Rheumatology key messages

- Stem cell transplantation trials with overlapping eligibility criteria may recruit different populations, which requires caution when comparing results.
- Stem cell transplantation inclusion criteria identify patients with unfavourable long-term outcomes despite current best practice immunosuppressive treatment.
- Trial exclusion criteria are valid, but our findings emphasize that better treatment strategies are needed for poor outcome patients.

## Introduction

SSc is a rare autoimmune rheumatic disease associated with inflammation and fibrosis of skin and internal organs, and widespread vasculopathy [1]. A subset of patients develops severe progressive disease, which is associated with high morbidity and mortality. Autologous haematopoietic stem cell transplantation (SCT) has emerged as a promising and effective treatment for these poor prognosis patients based upon the results of two large clinical trials [ASTIS (Autologous Stem Cell Transplantation International Scleroderma) and SCOT (Scleroderma: Cyclophosphamide Or Transplantation)] that demonstrated long-term benefits with regard to survival, skin involvement, lung function and quality of life [2, 3]. Additionally, a third randomized trial (the UPSIDE trial) is currently ongoing, investigating SCT in early dcSSc (NCT04464434) [4]. The use of intensive treatment regimens in SCT carries also an increased risk of severe complications [5, 6]. Inclusion criteria for SCT trials in SSc aim to recruit cases with high risk for disease progression, while excluding patients with more severe organ-based disease that would compromise potential success of the treatment. Exclusion criteria are particularly selected to minimize treatment-related mortality.

Over the past two decades the understanding of disease mechanisms and routine management in SSc have improved and use of DMARDs such as MMF are increasingly administered in the early course of the disease [7–10]. These developments may have improved survival of cases that would have been eligible for SCT trials compared with historically predicted outcomes.

The aim of this study was to explore morbidity and mortality in a real-world cohort of SSc patients fulfilling eligibility criteria for ASTIS, SCOT and the ongoing UPSIDE study, but who have been treated with standard immunosuppression, with long-term outcome data available. This provides insight into long-term outcomes with contemporary standard treatment and highlights the value of careful case selection for SCT.

## Methods

### Patient selection

Patients with disease onset, defined by first non-Raynaud SSc manifestation, between 1980 and 2020 were selected from the Royal Free London ScleroderMA cohoRT (SMART) using ASTIS, SCOT and UPSIDE (UPfront autologous haematopoietic Stem cell transplantation *vs* Immunosuppressive medication in early DiffusE cutaneous systemic sclerosis) trial inclusion criteria.

ASTIS inclusion criteria used to select patients for this study were: age 16–65 years, diffuse subtype of disease, modified Rodnan skin score (mRSS) ≥20 or mRSS ≥15, and major organ involvement [diffusing capacity of the lungs for carbon monoxide (DLco) and/or forced vital capacity (FVC) ≤80% and high-resolution CT (HRCT) abnormalities/renal/cardiac disease] and disease duration <4 years after onset non-RP symptoms [2]. Exclusion criteria were age >65 years, pulmonary hypertension (PH), DLco <40% and/or estimated creatinine clearance <40 ml/min. We were not able to use systolic ejection fraction at baseline to exclude patients due to missing data.

SCOT inclusion criteria used to select patients were: age 18–65 years, diffuse subtype of disease, mRSS ≥16 and disease duration <4 years after onset non-RP symptoms and either SSc-related pulmonary disease with DLco and/or FVC ≤70 % and/or a history of renal disease [3]. Exclusion criteria were age >65 years, PH, DLco <40% or FVC <45% of predicted, and estimated creatinine clearance <40 ml/min. UPSIDE trial inclusion criteria are: age 18–65 years, diffuse subtype of disease and disease duration <2 years after onset non-RP symptoms, and either mRSS ≥15 and/or SSc-related pulmonary disease with DLco and/or FVC ≤85% or cardiac or renal disease [4].

Exclusion criteria were age >65 years, PH and DLco <40% of predicted and/or estimated creatinine clearance <40 ml/min. Cardiac disease was defined as SSc-related congestive heart failure, rhythm disturbances or pericardial effusion, and was an inclusion criterium for the ASTIS and UPSIDE trial. Patients with cardiac involvement were excluded from participation in the SCOT trial. (Table 1).

**Table 1:** Inclusion criteria from stem cell transplantation trials

ASTIS (2014)	SCOT (2018)	UPSIDE (2020)
Inclusion criteria		
**Age 16–65 years**	**Age 18–65 years**	**Age 18–65 years**
**dcSSc** ^a^	**dcSSc** ^a^	**dcSSc** ^b^
**Disease duration <4 years from non-RP**	**Disease duration ≤4 years from non-RP**	**Disease duration ≤2 years from non-RP**
**mRSS >20 +** ESR >25 mm + Hb 11 g/dl	**mRSS ≥16**	**mRSS ≥15**
or:	and at least one:	and/or:
**mRSS >15 + DLco and/or FVC ≤80% +** HRCT abnormalities	**FVC <70% or DLco <70%** + HRCT abnormalities	**DLco and/or FVC ≤85% and** HRCT abnormalities **or FVC decline of >10% or DLco decline of >15% within 12 months**
	**Renal involvement**	**and/or**
**and/or**		**Renal or cardiac involvement**
**Renal or cardiac involvement**		
Exclusion criteria		
**PAH**	**PAH**	**PAH**
LVEF <45%	LVEF < 50%. Pacemaker/ICD	LVEF <45%
**DLco <40%**	**DLco < 40%**	**DLco <40%**
**Creatinine clearance <40 ml/min**	**FVC <45%**	Previous treatments with MMF, MTX, AZA, RTX, steroids >6 months
	**Creatinine clearance <40 ml/min**, active SRC	Previous CYC
	Previous i.v. CYC >6 months (>4 months oral CYC)	Zubrod-ECOG-WHO Performance Status Scale >2
	Active GAVE	
	Active hepatitis	

The criteria shown in bold were used to select patients for this study.

a According to ARA-Classification Criteria Systemic Sclerosis.

b According to 2013 ACR-EULAR classification criteria for diffuse cutaneous SSc. DLco: diffusing capacity of the lungs for carbon monoxide; ECOG: Eastern Cooperative Oncology Group; FVC: forced vital capacity; GAVE: gastric antral vascular ectasia; Hb: haemoglobin; HRCT: high-resolution CT; ICD: implantable cardioverter-defibrillator; LVEF: left ventricular ejection fraction; mRSS: modified Rodnan skin score; PAH: pulmonary arterial hypertension; RTX: rituximab; WHO: World Health Organization.

All included patients provided written informed consent for the Royal Free London SMART project.

### Data collection

All clinical data pertaining to patient management were routinely collected from electronic patient records in a standardized manner as part of the patient’s routine visits for clinical assessment at the national referral centre for scleroderma at the Royal Free Hospital, London, UK. The patient records were the primary source of data for this study. Survival status was checked with the patients’ general practitioners in the event that they did not attend follow-up visits within 1 year. mRSS, FVC and DLco % predicted were collected at baseline, and at 1, 5, 10 and 15 years of follow-up. For data at baseline and 1 year, a window of 6 months was accepted, at 5 years follow-up this was 1 year, and at 10 and 15 years, data within 2 years were included. Cardiac involvement was defined as haemodynamically significant cardiac arrhythmias, conduction defects requiring pacemaker or implantable cardioverter-defibrillator, pericardial effusion or congestive heart failure. Severe pulmonary fibrosis (PF) was defined as >20% on HRCT extent and/or FVC <70% of predicted. Renal involvement was defined as scleroderma renal crisis (SRC), defined as new onset hypertension (>150 mmHg systolic blood pressure and diastolic blood pressure >85 mmHg) and documented decrease in estimated glomerular filtration rate >30%, or severe chronic kidney disease grade 4 or 5 including the need for renal replacement therapy due to causes other than SRC. PH was defined as having a mean pulmonary arterial pressure >25 mmHg and pulmonary capillary wedge pressure ≤15 mmHg confirmed by right heart catheterization. Baseline left ventricular ejection fraction (LVEF), ESR, haemoglobin and serum creatinine were included when they were done within the first year after diagnosis. The estimated creatinine clearance was calculated using the Modification of Diet in Renal Disease equation. Antibodies were collected and categorized in SSc-specific antibodies (anti-topoisomerase, anti-RNA-polymerase III, anti-U3 RNP and ANA). In cases where patients were positive for more than one antibody, patients were included in the antibody group specific to SSc. Antibodies ACA, SL, U1 RNP, nRNP, PM/Scl, SM, Ro, La, Ku and Th/To were categorized as ‘other’.

### Data analysis

Descriptive statistics were used to describe patient characteristics. Characteristics of groups were compared using χ^2^ tests for categorical data, the independent *t*-test in case of normally distributed data or the Mann–Whitney *U* test for non-parametric data. Characteristics of the three study groups were described only, as overlap between groups limited the use of statistical tests for comparisons. Overall survival and event-free survival (EFS) of patients eligible for one or more of the SCT studies were assessed using Kaplan–Meier (KM) survival estimates. Hazard ratios (HR) were calculated using Cox proportional hazards regression analysis. Proportionality of hazards assumption was tested for all explanatory variables by analysing the residuals and log-log plots, and we included time-varying effects in the regression models. Overall survival was defined as the time in years from eligibility for one of the SCT trials until death. EFS in this study was defined as the time in years from eligibility for one of the SCT trials until the occurrence of death due to any cause or the development of persistent major organ event, defined as severe cardiac involvement, severe PF, SRC or PH.

The effect of smoking, age at onset, sex, autoantibodies, ESR, haemoglobin, creatinine, mRSS, LVEF, FVC and DLco % of predicted at baseline, the period of diagnosis and medication use on the hazards was assessed using Cox proportional hazards regression analysis. Factors with an association with *P* < 0.1 were entered in a multivariable Cox regression analysis. Cumulative incidence of organ complications (cardiac, renal, PF and PH) at 2, 5, 10 and 20 years of follow-up were calculated using the 1-KM. Changes in lung function tests (FVC % of predicted, DLco % of predicted) and mRSS over time were assessed using linear mixed-effects models. SPSS version 25 was used for all analyses.

This project was conducted in compliance with the declaration of Helsinki and was approved by the London-Hampstead NRES Committee (MREC Reference ID 6398) and London‐Fulham Research Ethics Committees (IRAS project ID 279682) for later cases.

## Results 

**Fig. 1:**
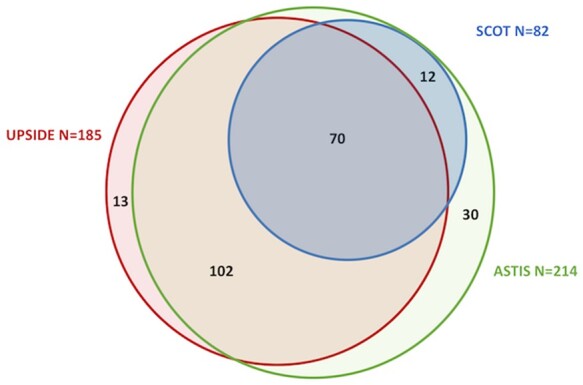
Venn diagram showing overlap of patients eligible for stem cell transplantation trials ASTIS: Autologous Stem Cell Transplantation International Scleroderma; SCOT: Scleroderma: Cyclophosphamide Or Transplantation; UPSIDE: UPfront autologous haematopoietic Stem cell transplantation vs Immunosuppressive medication in early DiffusE cutaneous systemic sclerosis.

Of the 636 patients with dcSSc in our cohort, 227 would have been eligible for one or more of the SCT trials. In all, 214 met the inclusion criteria for the ASTIS trial, 82 for the SCOT trial and 185 for the UPSIDE trial; 66 patients met the inclusion criteria for all three studies, but met also one or more exclusion criteria (ASTIS and SCOT *n* = 60 and UPSIDE *n* = 56). Patients were not eligible for the study because of age (ASTIS *n* = 18, SCOT *n* = 19, UPSIDE *n* = 22), low DLco (*n* = 20 in all studies), creatinine clearance <40 ml/min (*n* = 22 in all studies) or PH diagnosed within the first 2 or 4 years after diagnosis (dependent on the criteria of the study) (ASTIS and SCOT *n* = 8, UPSIDE *n*  = 1) (supplementary Fig. S1, available at *Rheumatology* online). There was overlap between patient groups that were eligible for participation (Fig. 1). Patient characteristics are shown in Table 2.

**Table 2:** Patient characteristics

Characteristics	Eligible, *N* = 227	Excluded^c^, *N* = 66	*P*-value^d^	ASTIS eligible^b^, *N* = 214	SCOT eligible, *N* = 82	UPSIDE eligible, *N* = 185
Age at onset in years [mean (s.d.)]	44.6 (11.6)	52.9 (53.0)	**<0.001**	44.6 (11.5)	44.5 (12.6)	45.8 (11.0)
Disease duration at time of inclusion in years (median^a^)	1.1 (0.8–1.7)	1.4 (0.8–2.6)	0.070	1.1 (0.8–1.7)	1.1 (0.9–1.7)	1.0 (0.7–1.5)
Onset [*n* (%)]						
1980–99	93 (41.0)	19 (28.8)	0.073	90 (42.1)	37 (45.1)	72 (38.9)
2000–20	134 (59.0)	47 (71.2)		124 (57.9)	45 (54.9)	113 (61.1)
Female [*n* (%)]	178 (78.4)	47 (71.2)	0.223	169 (79.0)	63 (76.8)	144 (77.8)
Follow-up time in years (mean, s.d.)	12 (7)	9 (6)	**<0.001**	13 (7)	12 (7)	12 (7)
mRSS at inclusion [mean (s.d.)]	26.1 (9.4)	24.0 (9.0)	0.116	26.4 (9.3)	25.6 (9.3)	25.8 (9.5)
FVC % pred at inclusion [mean (s.d.)]	85.8 (17.3)	83.1 (25.3)	0.434	85.8 (17.3)	75.5 (16.0)	86.2 (16.9)
DLco % pred at inclusion [mean (s.d.)]	69.8 (17.0)	55.6 (20.2)	**<0.001**	69.7 (17.1)	61.6 (16.6)	70.0 (16.7)
ESR, mm/h (first year) (median)	12.0 (6–24)	21.0 (8–48)	**0.006**	13.0 (7–24)	14.0 (7–27)	13.0 (7–24)
Hb, g/dL (first year) [mean (s.d.)]	11.6 (2.1)	11.7 (2.2)	0.661	11.7 (2.2)	12.0 (1.8)	11.7 (2.1)
Creatinine, μmol/l (first year) (median)	67.0 (57–79)	91.0 (64–227)	**<0.001**	67.0 (58–79)	67.0 (57–86)	67.5 (59–80)
LVEF, % (first year) (median)	65.0 (60–65)	60 (55–65)	0.125	65.0 (60–65)	65.0 (60–65)	65.0 (60–65)
Autoantibodies [*n* (%)]			0.178			
ATA	71 (31.7)	25 (38.5)		70 (33.2)	35 (43.2)	58 (31.7)
ARA	72 (32.1)	22 (33.8)		69 (32.7)	20 (24.7)	64 (35.0)
Anti-U3RNP	14 (6.3)	3 (4.6)		12 (5.7)	4 (4.9)	8 (4.4)
Other	58 (25.9)	12 (18.5)		52 (24.6)	17 (21.0)	44 (24.0)
ANA negative	9 (4.0)	3 (4.6)		8 (3.8)	5 (6.2)	9 (4.9)
Smoking, *n* (%)			0.097			
Never	106 (46.7)	20 (47.6)		101 (51.0)	42 (59.2)	88 (50.6)
Ever	103 (45.4)	23 (52.4)		97 (49.0)	29 (40.8)	86 (49.4)
Medication use (ever) [*n* (%)]						
MTX	52 (22.9)	12 (27.9)	0.489	50 (25.4)	16 (22.9)	46 (26.9)
MMF	150 (66.1)	24 (55.8)	0.085	141 (71.6)	51 (72.9)	124 (72.5)
CYC	48 (21.2)	10 (23.2)	0.353	45 (22.8)	16 (22.9)	41 (24.0)
RTX	5 (2.2)	1 (2.2)	0.577	4 (2.0)	2 (2.9)	5 (2.9)

Bold text represents significant *P*-values (*P* < 0.050).

a For all median values, 25–75th percentiles are reported.

b There was overlap between patient groups that were eligible for participation to the three trials (see also Fig. 1).

c Patients met criteria of exclusion from at least one the SCT trials.

d Independent samples *t*-test, Pearson χ^2^ test or Mann–Whitney *U* test. ATA: anti-topoisomerase antibodies; ARA: anti-RNA polymerase III antibodies; ASTIS: Autologous Stem Cell Transplantation International Scleroderma; DLco: diffusing capacity of the lungs for carbon monoxide; FVC: forced vital capacity; Hb: haemoglobin; LVEF: left ventricular ejection fraction; mRSS: modified Rodnan skin score; RTX: rituximab; SCOT: Scleroderma: Cyclophosphamide Or Transplantation; UPSIDE: UPfront autologous haematopoietic Stem cell transplantation *vs* Immunosuppressive medication in early DiffusE cutaneous systemic sclerosis.

Patients meeting the exclusion criteria from all SCT trials had a significantly higher age at onset (*P* < 0.001), shorter follow-up time (*P* < 0.001), lower baseline DLco % of predicted (*P* < 0.001), higher creatinine at baseline (*P* < 0.001) and a higher ESR in the first year of diagnosis (*P* = 0.006).

### Outcomes

#### Survival

During a mean follow-up of 12 years (s.d. 7), 103 of the eligible patients (45.4%) died. Median age at death was 60 years (Q1–Q3: 52–67). The number of deaths was significantly higher in the group of excluded patients (71.2%, *P* < 0.001) (supplementary Table S1, available at *Rheumatology* online).

Survival among the 226 available and eligible patients was 96% at 2, 88% at 5, 73% at 10 and 43% at 20 years. The 66 patients who fulfilled inclusion criteria but would have been excluded because of severe organ disease or age had a worse survival of 97% at 2, 77% at 5, 52% at 10 and 15% at 20 years (log rank *P* < 0.001) (Fig. 2.1A).

**Fig. 2:**
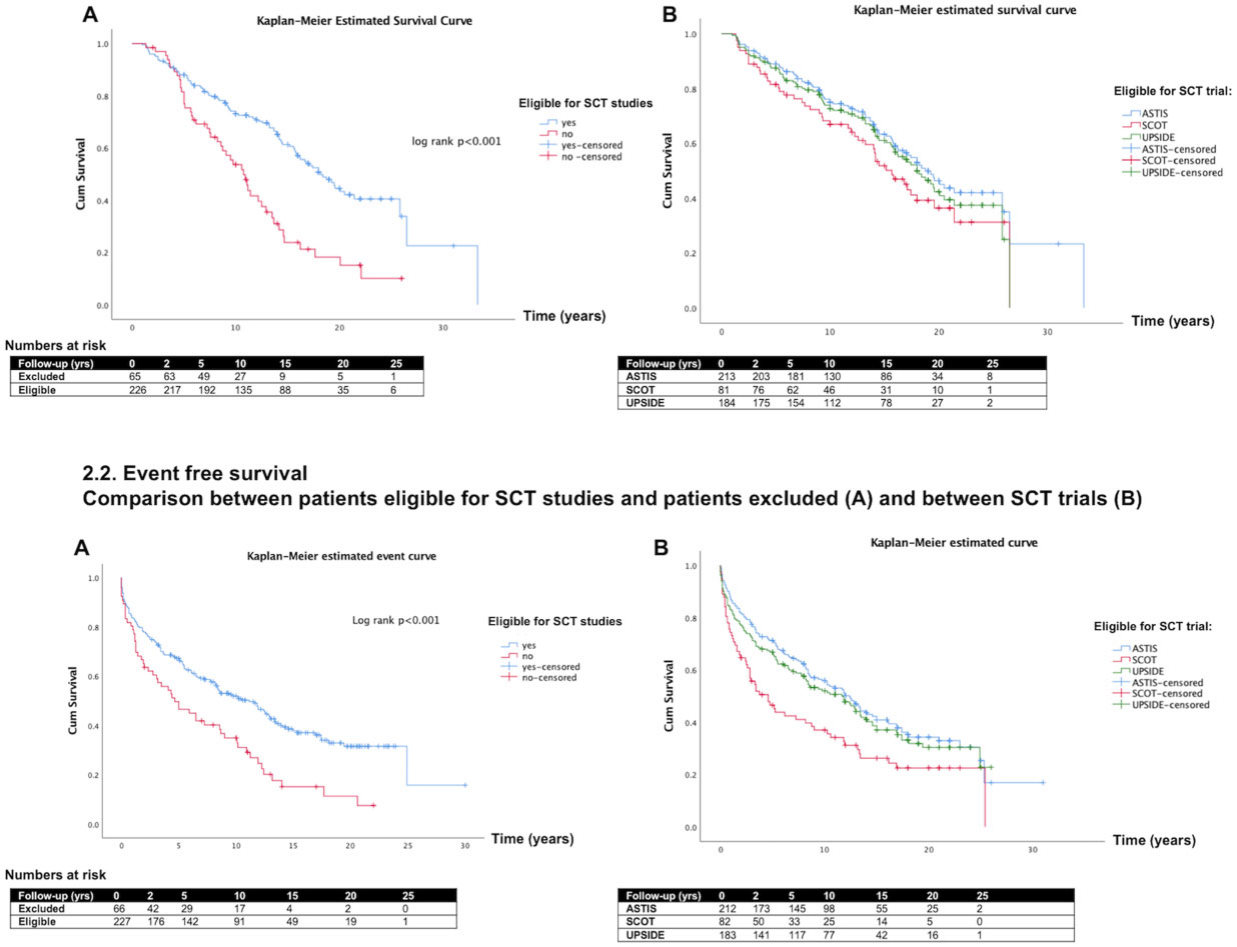
Survival curves overall (2.1) and event-free survival (2.2) ASTIS: Autologous Stem Cell Transplantation International Scleroderma; SCT: stem cell transplantation; SCOT: Scleroderma: Cyclophosphamide Or Transplantation; UPSIDE: UPfront autologous haematopoietic Stem cell transplantation vs Immunosuppressive medication in early DiffusE cutaneous systemic sclerosis

Survival among patients eligible for the ASTIS trial was 96% at 2, 88% at 5 and 75% at 10 years. For SCOT this was 93% at 2, 80% at 5 and 64% at 10 years, and 95% at 2, 87% at 5 and 72% at 10 years for UPSIDE (Fig. 2.1B). Multivariable analysis demonstrated that hazard of death was higher in patients with higher age at onset (HR 1.05, *P* < 0.001, 10-year increase in age increased the hazard of death by 63%), higher ESR at baseline (HR 1.01, *P* = 0.025, for every 10-mm higher ESR the risk of death increased by 10%) and males (HR 2.12, *P* = 0.008). Higher DLco % predicted at inclusion was associated with a lower hazard of death (HR 0.98, *P* = 0.008, for 10% higher DLco predicted at baseline the risk of death falls by 18%) (Table 3).

**Table 3:** Univariable and multivariable associations (Cox regression analysis) for overall survival in patients eligible for SCT trials

	Univariable	*P*-value	Multivariable^a^	*P*-value
	HR (95% CI)		HR (95% CI)	
Age at onset	1.04 (1.02, 1.06)	<0.001	1.05 (1.03, 1.08)	**<0.001**
Time period of onset				
Referent: 2000–20				
1980–99	1.02 (0.68, 1.54)	0.912		
Sex				
Referent: female				
Male	1.97 (1.30, 2.97)	0.001	2.12 (1.22, 3.71)	**0.008**
mRSS at inclusion	1.03 (1.01, 1.06)	0.005		
FVC at inclusion	0.97 (0.96, 0.98)	<0.001		
DLco at inclusion	0.97 (0.96, 0.98)	<0.001	0.98 (0.96, 0.99)	**0.008**
Autoantibodies				
Referent: ATA				
ARA	0.47 (0.28, 0.80)	0.005		
Anti-U3RNP	0.72 (0.28, 1.83)	0.487		
Other	0.95 (0.58, 1.55)	0.828		
*Antibody × time interaction*				
ARA × time	0.52 (0.30, 0.90)	0.019		
Anti-U3RNP × time	0.50 (0.32, 2.31)	0.758		
Other	0.76 (0.97, 1.01)	0.446		
Smoking status				
Referent: never				
Ever	1.45 (0.94, 2.23)	0.091		
ESR (first year)	1.03 (1.01, 1.04)	<0.001	1.01 (1.00, 1.03)	**0.025**
Creatinine (first year)	1.00 (1.00, 1.02)	0.179		
Hb (first year)	1.06 (0.94, 1.18)	0.360		
MMF use ever	0.89 (0.55, 1.43)	0.632		
CYC use ever	1.33 (0.87, 2.14)	0.178		
MTX use ever	0.92 (0.57, 1.48)	0.723		

In bold are the significant *P*-values (*P* < 0.050) and *P*-values from variables included in the multivariable analysis (*P* < 0.100).

a Factors with an association with *P* < 0.100 in the univariable regression analysis were entered in the multivariable Cox regression analysis. Only the significant associations are shown. ATA: anti-topoisomerase antibodies; ARA: anti-RNA polymerase III antibodies: DLco: diffusing capacity of the lungs for carbon monoxide; FVC: forced vital capacity; Hb: haemoglobin; HR: hazard ratio; mRSS: modified Rodnan skin score; SCT: stem cell transplantation.

#### Events

The EFS (serious organ involvement or death) was 78% at 2, 66% at 5, 51% at 10 and 37% at 15 years in patients eligible for SCT trials. There was a significant difference in EFS between patients eligible and patients excluded for SCT trials (log rank *P* < 0.001) (Fig. 2.2). Male sex was an independent risk factors for an event (HR 1.97, 95% CI 1.34–2.88, *P* = 0.001) and DLco % predicted at baseline was associated with a lower hazard for a serious event (HR 0.98, 95% CI 0.97–0.99, *P* = 0.001, for 10% higher DLco predicted at baseline the risk of an event falls by 18%) in the multivariable Cox regression analysis (supplementary Table S2, available at *Rheumatology* online).

SRC was reported in 9 (4.0%) of eligible patients, 48 (21.1%) developed severe PF, 22 (9.7%) developed cardiac involvement and 23 (10.1%) PH. Patients excluded for the SCT trials more often developed PH (19.7%, *P* = 0.037) compared with eligible patients, while patients eligible for the trials more often developed PF (34.3% *vs* 18.2%, *P* = 0.012). The time to severe PF, PH and SRC was significantly shorter in patients excluded for SCT trials. The occurrence of (severe) PF and SRC and the time to severe PF differed between SCT trials. Patients fulfilling the SCOT trial had more often and earlier severe lung involvement compared with the other trials. Onset of PH and SRC was later in patients eligible for the ASTIS trial (supplementary Table S1, available at *Rheumatology* online).

#### Changes of skin and lung function over time in eligible patients

**Fig. 3:**
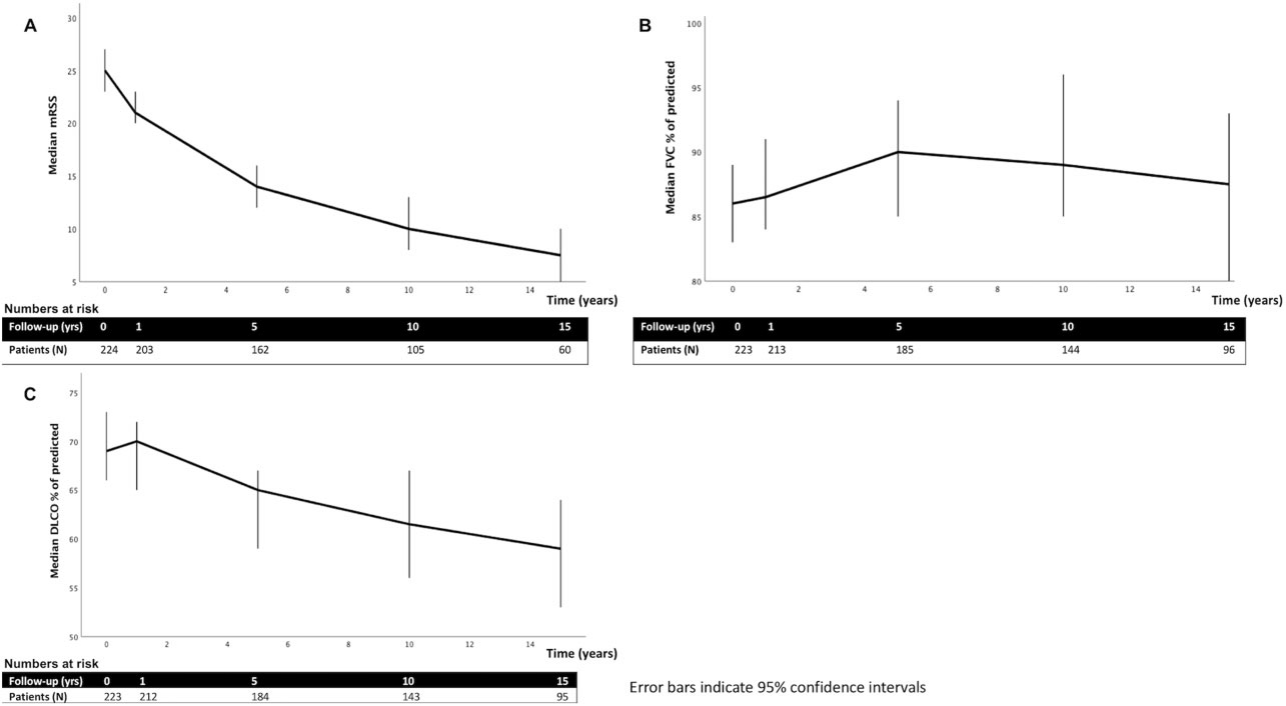
Course of mRSS, FVC and DLco over time in patients eligible for SCT trials DLco: diffusing capacity of the lungs for carbon monoxide; FVC: forced vital capacity; mRSS: modified Rodnan skin score.

The median mRSS, FVC % of predicted and DLco % of predicted during follow-up in patients eligible for SCT trials are shown in Fig. 3. The mean predicted mRSS gradually declined with a nonlinear course (mRSS = 25.7–2.1 × years + 0.06 × years^2^, *P* < 0.001 for both parameters). The mean estimated average change in mRSS from baseline to 1 year was −2.2 units, and –12.1 units between baseline and 5 years in patients eligible for SCT studies. There was a nonlinear change in the estimated average of FVC % predicted (FVC = 85.6–0.8 × time + 0.2 × time^2^ – 0.01 × time^3^, *P* = 0.025, *P* < 0.001 and *P* < 0.001, respectively). So, the FVC was −0.6% between baseline and 1 year, and –0.1% between baseline and 5 years. The mean estimated average change in DLco % predicted was −1.7% between baseline and 1 year, and −7.7% between baseline and at 5 years (DLco = 69.8–1.7 × time + 0.03 × time^2^, *P* < 0.001 and *P* = 0.013, respectively).

## Discussion

In this study we explored morbidity and mortality in the observational SMART cohort of patients with dcSSc fulfilling the eligibility criteria for ASTIS, SCOT and UPSIDE trials, but who have been treated with standard immunosuppression. In this report, we present an analysis of the survival trajectory and pattern of organ involvement of a contemporaneous cohort of dcSSc treated with standard immunosuppression to validate the inclusion/exclusion criteria for the recent SCT trials.

We observed that patients in our cohort meeting the inclusion criteria of the SCT trials had poor outcomes despite current best practice treatment including routine early administration of MMF. Compared with patients treated with SCT in SCOT [3] and ASTIS [2] and the recently published Dutch observational SCT study [11], long-term overall survival was just marginally lower. EFS, however, was worse in our cohort. As severe organ involvement is associated with poor quality of life, we believe that our study justifies the place of SCT in dcSSc. Our data also confirmed that benefit of SCT on the short-term is less apparent compared with outcomes from 10 years’ follow-up. (supplementary Table S3, available at *Rheumatology* online). Survival of our cohort was better than the SCOT and ASTIS CYC-control arms.

This may possibly reflect the beneficial effect of MMF in dcSSc, which is consistent with other studies [9, 10]. Another possible explanation for the difference in outcomes could be that our cohort reflects a patient group with milder disease. Baseline characteristics of the CYC groups in ASTIS and SCOT had a lower mean DLco % predicted compared with our cohort (58% and 53%, compared with 69% in our cohort).

Furthermore, the use of different outcomes should be taken into account when comparing between cohorts. We were not able to use the global rank composite score used as the primary outcome in SCOT in our analysis [3]. Also, we used a slightly different definition for EFS, which reflects clinically meaningful organ complications affecting quality of life, rather than only permanent organ failure as was used in the trials. Although long-term overall survival is better in SCT trials compared with conventional immunosuppressants, severe adverse events related to transplantation are more often reported and short-term treatment-related mortality is higher [2, 3]. These aspects should be considered when deciding on the optimal treatment strategy.

Our study showed that eligibility criteria of SCT trials may substantially influence group-level outcome. We found that SCOT was the most restrictive study, including only patients with extensive lung disease or history of renal disease, while the currently ongoing UPSIDE trial allows dcSSc patients in the early phases to participate. By including patients with worse prognosis, SCOT may have shown benefits in a smaller trial and not necessarily achieved a larger effect compared with the ASTIS trial. Thus, these findings prompt caution when comparing treatment effects of trials.

Our study identified predictors for survival and serious organ complications. In the patients eligible for SCT, age at onset, male sex, DLco and ESR at baseline were associated with death, and male sex and DLco at baseline were associated with serious events. These findings are consistent with other studies reporting on prognostic factors. In the Dutch SCT cohort age, but also male sex, higher HCT-CI (SCT-specific comorbidity index) and LVEF <50% at baseline, were associated with lower EFS [11]. Smoking was not independently associated with survival in our cohort and the Dutch SCT cohort, contrary to what was observed in the ASTIS and SCOT trials [2].

In non-SCT studies, age at disease onset, anti-topoisomerase positivity and presence of PH were reported as risk factors for poor survival [12–14]. In our cohort, we did not observe differences in overall survival or EFS between antibody profiles. One explanation could be that we selected a high-risk population. In addition, events were counted after patients became eligible for one or more of the three trials, which means some patients already had severe PF, cardiac or renal disease.

Another main finding is the significantly worse survival in patients who would have been excluded for the SCT trials. Current exclusion criteria for SCT used in both clinical practice and trials are valid as these prevent exposing patients with very high risks for complications to SCT [15]. However, this leaves a group of worse-outcome patients without suitable treatment options. More research investigating less toxic SCT regimens or new targeted biologics are therefore needed to refine strategies for optimal management.

Our study has some limitations. First, because of the retrospective design there were missing data with regard to serial lung function and mRSS. We realize that there is a risk that the serial data presented could be biased as surviving patients presumably have better lung function values. We were able to minimize the number of missing data though, especially with regard to survival status. Second, comparisons between treatments and outcomes of SCT trials need to be interpreted with caution because of the risk of confounding by indication in our cohort with regard to treatment choices, and because of variation in patient groups and outcome measures. A strength of our study is the large number of included patients and the extensive data collection of the SMART cohort.

In conclusion, our findings confirm that SCT inclusion criteria identify patients with poor outcome and long-term EFS favours SCT. There remains a high unmet need for safe and effective treatment strategies in patients who are non-SCT-eligible due to age and extensive disease, as they appear to have a particularly poor prognosis. More research is needed to develop suitable therapeutic options for this group.

## Supplementary Material

keab604_Supplementary_Data
