# Supplementary material for: Outcomes linked to eligibility for stem cell transplantation trials in diffuse cutaneous systemic sclerosis
Source: Rheumatology (Oxford). 2021 Jul 26;61(5):1948–56. doi: 10.1093/rheumatology/keab604 (PMC9071533; doi:10.1093/rheumatology/keab604)
Supplement: keab604_Supplementary_Data [file keab604_supplementary_data.docx]

**Supplementary Table S1. Overview of events and death in the patients eligible and excluded for the SCT trials**

| **Events** | **Eligible**  **N= 227** | **Excluded**  **N=66** | p-value* | **ASTIS eligible**  **N=214** | **SCOT eligible**  **N=82** | **UPSIDE eligible**  **N=185** |
| --- | --- | --- | --- | --- | --- | --- |
| Pulmonary fibrosis, n (%) Onset (yrs, median (Q1-Q3) | 78 (34.4)  1.8 (1-5) | 12 (18.2) 1.2 (0-4) | **0.012**  0.249 | 73 (34.4)  1.8 (0-5) | 33 (40.2)  0.80 (0-2) | 69 (37.5) 2.0 (1-5) |
| Severe PF, n (%)  Onset (yrs, median (Q1-Q3)) | 48 (21.1)  4.5 (1-11) | 13 (19.7)  1.3 (0-2) | 0.799  **0.006** | 45 (21.2)  4.5 (1-10) | 27 (33.3)  1.4 (1-5) | 44 (23.8)  4.5 (1-11) |
| PH, n (%)  Onset (yrs, median (Q1-Q3)) | 23 (10.1)  5.5 (3-12) | 13 (19.7)  2.7 (1-5) | **0.037**  **0.012** | 18 (8.5)  7.5 (5-13) | 8 (10.0)  6.5 (4-12) | 19 (10.3)  5.5 (3-12) |
| Cardiac, n(%)  Onset (yrs, median (Q1-Q3)) | 22 (9.7)  3.5 (2-7) | 7 (10.6)  1.3 (1-3) | 0.827 0.199 | 23 (10.8)  3.5 (2-7) | 10 (12.2)  3.3 (0-6) | 19 (10.3)  4.5 (2-8) |
| SRC, n (%)  Onset (yrs, median (Q1-Q3)) | 9 (4.0)  1.5 (0-5) | 4 (6.1)  0.6 (0-0.2) | 0.467  **0.011** | 8 (3.8) 1.5 (0-5) | 4 (4.9)  1.3 (0-4) | 8 (4.3)  1.3 (0-4) |
| Death, n (%)  Onset (yrs, median (Q1-Q3)) | 103 (45.4)  9.2 (5-15) | 47 (71.2)  8.3 (5-12) | **<0.001** 0.155 | 104 (45.4)  9.5 (5-16) | 56 (58.3)  9.3 (4-14) | 89 (48.5)  9.4 (5-16) |

* Independent Samples T Test, chi-square test or Mann-Whitney U test.

Bold text represents significant p-values (p<0.050).

*ASTIS, Autologous Stem Cell Transplantation International Scleroderma; IQR, interquartile range; PF, pulmonary fibrosis; PH, pulmonary hypertension; SCOT, Scleroderma: Cyclophosphamide Or Transplantation; SCT, stem cell transplantation; SRC, scleroderma renal crisis; UPSIDE, UPfront autologous hematopoietic Stem cell transplantation versus Immunosuppressive medication in early DiffusE cutaneous systemic sclerosis; yrs, years.*

**Supplementary Table S2. Univariable and multivariable associations (cox regression analysis) for event free survival in patients eligible for SCT trials.**

|  | **Univariable**  **HR (95% CI)** | **p-value** | **Multivariable***  **HR (95% CI)** | **p-value** |
| --- | --- | --- | --- | --- |
| Age at onset | 1.01 (1.00-1.02) | **0.042** |  |  |
| Time period of onset  Referent: 2000-2020  1980-1999 | 1.00 (0.74-1.35) | 0.979 |  |  |
| Sex. Referent: Female  Male | 2.27 (1.56-3.29) | **<0.001** | 1.97 (1.34-2.88) | **0.001** |
| Mean mRSS at inclusion | 1.00 (0.99-1.02) | 0.606 |  |  |
| Mean FVC at inclusion | 0.98 (0.97-0.99) | **<0.001** |  |  |
| Mean DLco at inclusion | 0.98 (0.97-0.99) | **<0.001** | 0.98 (0.97-0.99) | **0.001** |
| Autoantibodies  Referent: ATA  ARA  Anti-U3RNP  Other  *Antibody x time interaction*  ARA x time  Anti-U3RNP x time Other x time | 0.47 (0.30-0.74)  0.84 (0.40-1.76)  0.74 (0.48-1.16)  0.48 (0.30-0.75)  0.84 (0.39-1.43)  0.76 (0.41-1.43) | **0.001**  0.639  0.190  **0.002**  0.664 0.396 |  |  |
| Smoking status Referent: never ever | 1.32 (0.97-1.79) | **0.079** |  |  |
| ESR (first year) | 1.01 (1.01-1.02) | **<0.001** |  |  |
| Creatinine (first year) | 1.00 (1.00-1.01) | **0.014** |  |  |
| Haemoglobin (first year) | 1.06 (0.98-1.15) | 0.145 |  |  |
| MMF use ever | 0.81 (0.59-1.12) | 0.204 |  |  |
| CYC use ever | 1.35 (0.96-1.90) | **0.082** |  |  |
| MTX use ever | 0.86 (0.61-1.23) | 0.417 |  |  |

*** Factors with an association with p <0.100 in the univariable regression analysis were entered in the multivariable Cox regression analysis. Only the significant associations are shown.

In bold are the significant p-values (p<0.050) and p-values from variables included in the multivariable analysis (p<0.100).

*ATA, anti-topoisomerase antibodies; ARA, anti-RNA polymerase III antibodies, CI, confidence interval; CYC, cyclophosphamide; DLco, diffusing capacity of the lungs for carbon monoxide; ESR, estimated sedimentation rate, FVC, forced vital capacity; HR, hazard rate; MMF, mycophenolate mofetil; mRSS, modified Rodnan Skin Score; MTX, methotrexate*

**Supplementary Table S3. Event free survival and overall survival of four cohorts**

| **Event Free survival** | **2yrs** | **5yrs** | **10yrs** | **15yrs** | **Overall survival** | **2yrs** | **5yrs** | **10yrs** | **15yrs** |
| --- | --- | --- | --- | --- | --- | --- | --- | --- | --- |
| **ASTIS** [2] |  |  |  |  | **ASTIS** |  |  |  |  |
| SCT arm | 82% | 81% | 65% |  | SCT arm | 85% | 84% | 74% |  |
| CYC arm | 86% | 67% | 54% |  | CYC arm | 83% | 74% | 55% |  |
| **SCOT** [3] |  |  |  |  | **SCOT** |  |  |  |  |
| SCT arm | 80% | 79% |  |  | SCT arm | 90% | 90% |  |  |
| CYC arm | 80% | 47% |  |  | CYC arm | 90% | 70% |  |  |
| **Dutch SCT cohort** [11] |  |  |  |  |  |  |  |  |  |
| SCT | 86% | 78% | 76% | 66% |  |  |  |  |  |
| **RFH-SMART cohort** |  |  |  |  | **RFH-SMART cohort** |  |  |  |  |
| Eligible for SCT | 78% | 66% | 51% | 37% | Eligible for SCT | 96% | 88% | 73% | 61% |
| Excluded for SCT | 62% | 45% | 33% | 11% | Excluded for SCT | 97% | 77% | 52% | 21% |
| Eligible for ASTIS | 81% | 71% | 55% | 40% | Eligible for ASTIS | 96% | 88% | 75% | 61% |
| Eligible for SCOT | 63% | 45% | 36% | 24% | Eligible for SCOT | 93% | 80% | 64% | 48% |
| Eligible for UPSIDE | 77% | 66% | 51% | 36% | Eligible for UPSIDE | 95% | 87% | 72% | 59% |

*ASTIS, Autologous Stem Cell Transplantation International Scleroderma; CYC, cyclophosphamide; EFS, event free survival; OS, overall survival; RFH-SMART, Royal Free London ScleroderMA cohoRT; SCOT, Scleroderma: Cyclophosphamide Or Transplantation; SCT, stem cell transplantation; UPSIDE, UPfront autologous hematopoietic Stem cell transplantation versus Immunosuppressive medication in early DiffusE cutaneous systemic sclerosis; yrs, years*


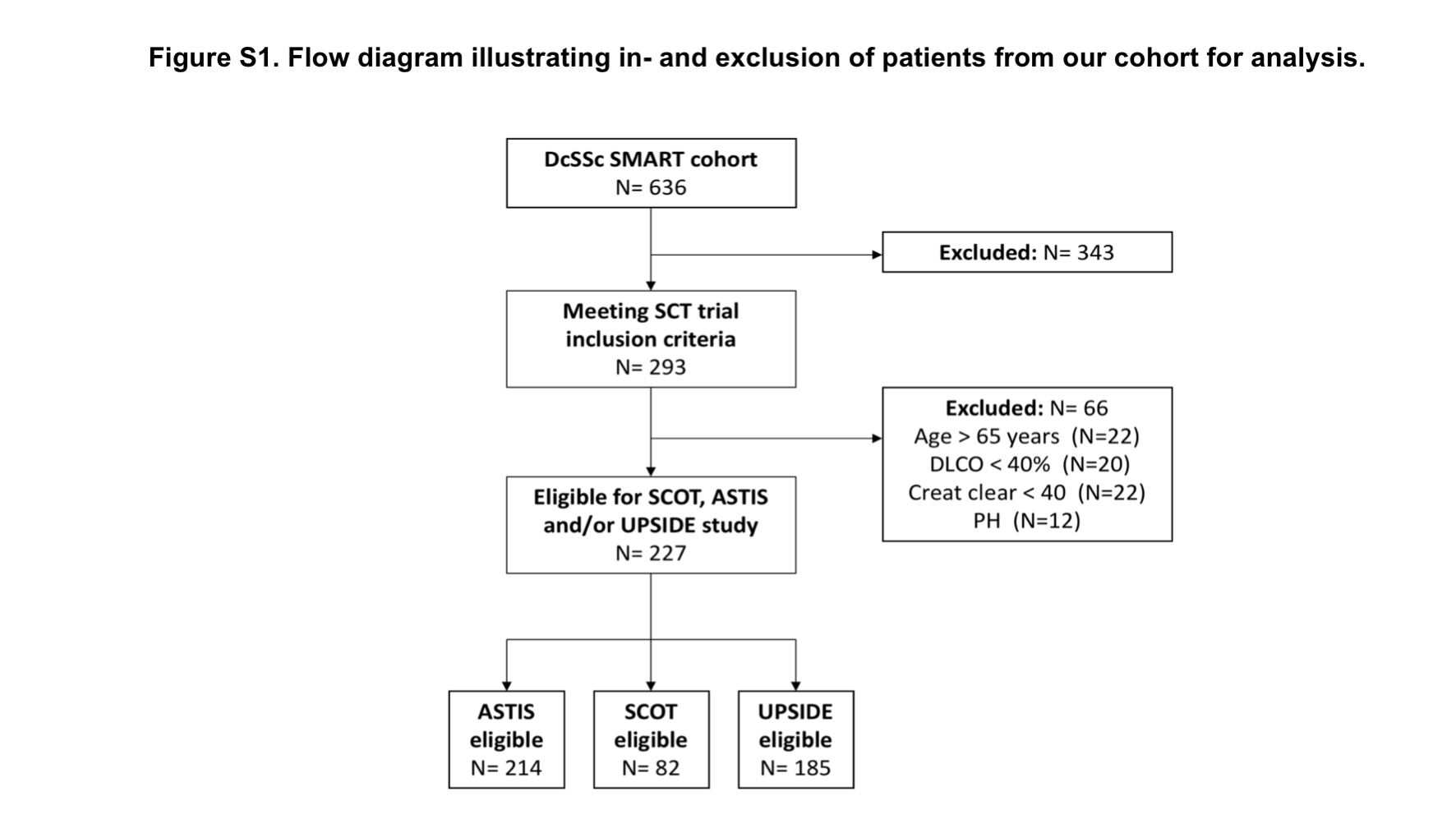


**Supplementary Figure S1. Flow diagram illustrating in- and exclusion of patients from our cohort for analysis**
